# Understanding Professional Medical Interpreters’ Perspectives on Advancing Accurate and Culturally Informed Patient–Provider Communication for Filipinos in Hawaiʻi: Qualitative Analysis

**DOI:** 10.3390/ijerph20217012

**Published:** 2023-11-02

**Authors:** Uliana Kostareva, Carrie A. Soo Hoo, Suzanne M. Zeng, Cheryl L. Albright, Clementina D. Ceria-Ulep, Holly B. Fontenot

**Affiliations:** 1 Nancy Atmospera-Walch School of Nursing, University of Hawai‘i at Mānoa, Honolulu, HI 96822, USA; 2 School of Education, Victoria University of Wellington, Kelburn, Wellington 6012, New Zealand; 3 Language Services Hawaii, 3747A Waialae Avenue, Honolulu, HI 96816, USA

**Keywords:** health equity, health literacy, health disparities, limited English proficiency, culturally and linguistically appropriate care, immigrants/migrants, medical interpreters

## Abstract

One in every eight persons in Hawaiʻi, USA, have limited English proficiency (LEP) and are entitled to free language assistance for federally funded services under Title IV of the Civil Rights Act of 1964. They also have the right to culturally and linguistically appropriate services (CLAS) provided by professional medical interpreters (PMIs). This study’s goals were to uncover barriers and facilitators of CLAS from the perspective of PMIs. PMIs for Filipino languages (*n* = 10) participated in an online survey and semi-structured interviews. Quantitative data were analyzed using descriptive statistics, and qualitative data were analyzed using conventional content analysis. Six themes emerged in the qualitative analysis: (1) cultural and social factors that can influence patient–provider communication; (2) barriers to effective patient–provider communication: patient, healthcare provider, and PMI levels; (3) facilitators of effective patient–provider communication: patient, healthcare provider, and PMI levels; (4) COVID-19 and remote interpreting barriers and facilitators; (5) strengths and weaknesses of in-person and stand-by interpreting appointments; and, (6) recommendations: system and provider levels. Proposed interventions could include advertising language services among Filipino communities and educating them about their language rights, providing additional resources for language assistance, employing more PMIs, training staff/providers, and supporting the use of PMIs versus untrained individuals.

## 1. Introduction

In the USA, almost 45 million people (13.6% of the population [1]) are immigrants [2]. Sixty-eight million speak a non-English language at home [3], and 9% of the total population (ages 5 and older) speak English less than “very well” and are considered as having limited English proficiency (LEP) [4]. Other terms include people who use language other than English (LOE) [5] and people with non-English language preference (NELP) [6].

There is a large and growing diaspora of immigrants from the Philippines living in the USA, representing nearly 5% of all immigrants (2 million) [7]. Hawaiʻi is one of the most diverse states in the USA where, in 2020, one in every five persons (18.2%) was foreign-born, and one in eight (12.4%) had LEP [4,8,9]. Of Hawaii’s 1.4 million residents, nearly 400,000 identified as Filipino, and a third (34%) of them were foreign-born [9,10]. Filipinos are the fastest-growing ethnic group in the state, with the Ilocano-speaking population tripling between 1980 and 2014 [11]. By 2014, Ilocano (17.6%) and Tagalog (17.6%) became the most spoken non-English languages at home [9]. Less than 40% of Ilocano and Tagalog speakers reported speaking English “very well” [9].

### 1.1. Relationship between Limited English Proficiency (LEP) and Health Literacy

For people with LEP, there are multiple health inequities and long-standing disparities in their healthcare [12,13]. LEP increases the risk of miscommunications with healthcare providers, decreases access to care, leads to poorer health outcomes, results in unsafe medical practices, and increases healthcare costs [14,15,16,17,18,19,20,21,22,23]. LEP can also impact an individual’s participation in informed medical decision making (e.g., consent, medication therapy), which, in turn, has been linked with personal health literacy—the ability to find, understand, and apply health information [16,24,25]. LEP is associated with lower personal health literacy, including among Asians and Pacific Islanders, which includes Filipinos [14,15,25]. Low health literacy and LEP are co-occurring barriers to safe and effective healthcare that contribute to lower medical comprehension, can influence health outcomes and patient compliance, and may lead to adverse events [14,15,16,24,25,26]. Both LEP and low health literacy have been highlighted as health disparities in Hawaiʻi exacerbated by the COVID-19 pandemic [27,28].

### 1.2. Definitions of Health Equity and Culturally and Linguistically Appropriate Services

Based on Title IV of the Civil Rights Act of 1964 and Presidential Executive Order 13,166 of 2000, patients are entitled to language assistance in both inpatient and outpatient settings [29]. Any medical facility receiving federal funds must provide free language assistance to patients with LEP [30]. The denial or delay of medical care due to language barriers is considered discrimination based on national origin [29]. In 2001, to advance health equity, improve care quality, and help eliminate healthcare disparities, the U.S. Department of Health and Human Services Office of Minority Health issued the National Standards for Culturally and Linguistically Appropriate Services (CLAS) in health and healthcare [31]. CLAS refers to “care and services that are respectful of and responsive to the cultural and linguistic needs of all individuals” [13] (p. 8). In 2006, the Hawaiʻi legislature passed Act 290, known as the Language Access law, to ensure that LEP individuals have equal and meaningful access to state-funded services [32].

### 1.3. Role of Professional Medical Interpreters

The provision of CLAS through language assistance via trained professional medical interpreters (PMIs) is critical for patient safety and effective patient–provider communication [23,33]. PMIs are trained to follow medical interpreting standards to help establish a clear line of communication and build rapport and trust between patient and provider [34]. In the USA, the medical interpreter certification can be obtained through the National Board of Certification for Medical Interpreters [35] and the Certification Commission for Healthcare Interpreters [36]. Research shows that many patients with LEP do not receive consistent PMI services and are assisted by ad hoc interpreters, such as untrained staff whose linguistic skills have not been assessed or patients’ friends or family members, including minors [22,37,38,39,40]. Compared to ad hoc interpreters, PMIs provide better quality interpreting and have fewer interpretation errors [41].

Knowledge gaps exist in the research focused on medical interpreter use, and there are few interventions addressing CLAS provision in healthcare [42,43]. Research on interpreters’ perspectives on CLAS provision either comes from Australia or the United Kingdom [44,45,46,47,48], which have different healthcare systems than the USA, or from the Midwest of the USA [34,49,50,51], which has limited applicability to Filipinos and Hawaiʻi. Furthermore, there is a dearth of research that explicates barriers and facilitators to CLAS from the perspective of PMIs. A PubMed review identified no publications in the English language reporting perspectives of PMIs who spoke Filipino languages.

Also, provision of CLAS by PMIs has been impacted by the COVID-19 pandemic [52]. Some studies reported decreases, while others reported increases in PMI use [53,54]. While in-person interpreting appointments halted, new opportunities emerged, including the incorporation of novel approaches and a transition toward more remote interpreting [54,55,56].

### 1.4. Study Objectives

The objective of this study was to help fill the current research gap by exploring perceived barriers and facilitators to providing CLAS for Hawaii’s Filipino LEP patients, also referred to as clients, from the perspective of PMIs. Our aims were to (1) explore context-specific factors that influence communication, such as culture; (2) uncover barriers and facilitators to CLAS provision; and (3) elicit recommendations to improve CLAS provision.

## 2. Materials and Methods

This study was conducted in collaboration with a community-based partner, Language Services Hawaiʻi (LSH), and used both quantitative and qualitative methods, i.e., surveys and interviews. Data were collected remotely via the Internet and in the English language.

### 2.1. Participants Recruitment and Procedures

Participants were recruited via a community partner (LSH), the largest provider of interpreter services in the state of Hawaiʻi. In 2022, LSH completed a total of 2914 medical interpreting appointments for Filipino languages. Eligibility criteria included working as a medical interpreter and access to a device with an internet connection or a phone. Potential participants’ contact information was obtained from LSH. Recruitment was conducted via email or phone. Interested PMIs selected a date and time for the interview and were directed to an online consent form and questionnaire via secured Google Forms.

Individual semi-structured interviews were completed one-on-one in November 2022. All interviews were conducted via Zoom^®^, except one, which was performed via phone due to connectivity issues. Participants (interviewees) and interviewer (principal investigator/first author) did not have previously established relationships; participants were made aware of the study goals during recruitment and via informed consent. Participants knew that the interviewer had a medical background, was an immigrant to the USA (not from the Philippines), and had previous experience working with PMIs in the medical setting. In addition, three key informant interviews with community partners were performed one-on-one between October 2022 and May 2023 via Zoom^®^ and phone.

The audio recordings of interviews were transcribed using Otter^®^ software version 2023. The accuracy of transcription was verified by the principal investigator and a research assistant (U.K. and C.S.H.). Each interviewee was given a pseudonym to protect confidentiality, and all identifiable recruitment data were stored securely and separately from other study data. Each participant was remunerated for their time with an 80 USD e-Gift card. The study was approved by the Institutional Review Board at the University of Hawaiʻi at Mānoa.

### 2.2. Measures

The questionnaire’s demographic data included age, number of years lived in the USA and Hawaiʻi, sex, race/ethnicity, country of birth, education, country(ies) where education was received, and level of education. Work experience-related data included interpreter training, experience with different interpreting appointment types (i.e., remote (phone or video), in-person, and stand-by), Filipino languages used for medical interpreting, months/years of experience as a PMI, and number of hours working as a PMI per week. Two Likert scale questions were “In your opinion, we need more trained Filipino interpreters in Hawaiʻi…” and “In your opinion, there are many healthcare appointments when an interpreter was needed, but none was provided…”. Response categories were as follows: agree completely, agree somewhat, disagree somewhat, disagree completely, and I do not know/no opinion/no experience.

Semi-structured interview questions were developed by three researchers (U.K., C.L.A., and H.B.F.) using the Health Disparities Research Framework model to capture key components related to the levels and domains of influence (PMI, patient, provider, and healthcare system). Questions were tailored to the study population with guidance from the Filipino cultural consultant (C.D.C.-U.) and community partner (S.M.Z.). See Table 1 for several examples of interview questions by domains of influence.

### 2.3. Analyses

All data were collected and managed using the principal investigator’s secure Google Forms and Sheets. Quantitative data were analyzed using descriptive statistics: mean, standard deviations, range for continuous data, and percentages for nominal data. Qualitative transcript data were analyzed using conventional content analysis [57,58]. Two investigators/coders (U.K. and C.A.S.H.) developed preliminary codes and a codebook. They coded six interviews together, discussed discrepancies, and refined the codes. Then, they coded the remaining four interviews independently but routinely met to review codes, categories, and emerging themes to ensure accuracy and validity. The definition of themes related to the Filipino culture was discussed with a Filipino cultural consultant, health professional, and co-author (C.D.C.-U.). In the event of a disagreement between the two coders, a qualitative researcher and senior health professional co-author (H.B.F.) was available to adjudicate the final codes.

## 3. Results

The collaboration with LSH facilitated access to the largest pool of PMIs for Filipino languages in the state. At the time of the study, there were 13 actively working PMIs for Filipino languages. The participation rate was 77% or 10 out of 13. Interviews ranged from 100 to 144 min. In February 2023, all interviewees received a written summary of their interview with an option to provide comments and have a follow-up call. One interviewee completed a 32 min follow-up call. In addition, there were three key informant interviews with the community partner.

### 3.1. Quantitative: Survey Results

The participants’ age range was from 32 to 67 years old, but most were in their 50 s or 60 s. The number of years working as a PMI ranged from 5 months to 24 years. Seven worked less than 20 h per week, and three worked more than 20 h per week as medical interpreters. Of the 10 participants, 80% identified as female; all participants were Filipino immigrants from the Philippines and were college graduates. Most (70%) PMIs completed two or more training modalities. Ilocano (70%) was the most common interpreting language among PMIs. A total of 7 out of 10 were interpreters for two or more Filipino languages. Many (60%) were experienced in all interpreting appointment types: in-person, video, phone, and stand-by. The overwhelming majority (90%) agreed that there was a need for more interpreters for Filipino languages, and 80% agreed somewhat/agreed completely that there were health appointments that occurred without a PMI present while a PMI was needed. See Table 2 for participants’ demographic information and PMIs’ opinions about the fulfillment of Filipino language interpretation needs in Hawaiʻi.

### 3.2. Qualitative Results

Interviewees reported having interpreted in various inpatient and outpatient settings: pre- and post-surgery, during hospitalizations, and in various departments of all major hospitals in the state, such as oncology, endocrinology, cardiology, neurology, ophthalmology, gastroenterology, at the transplant center, during physical therapy, and in the geriatric, pediatric, pre- and post-natal, and mental health settings.

Six main themes emerged from the qualitative analysis: (1) cultural and social factors that can influence patient–provider communication; (2) barriers to effective communication: patient, healthcare provider, and PMI levels; (3) facilitators of effective communication: patient, healthcare provider, and PMI levels; (4) COVID-19 and remote interpreting barriers and facilitators; (5) strengths and weaknesses of in-person and stand-by interpreting appointments; and (6) recommendations: system and provider levels. See Table A1 in Appendix A for additional illustrative quotes for these six themes.

#### 3.2.1. Cultural and Social Factors That Influence Patient–Provider Communication

The interviewees discussed several examples of how Filipino culture and family factors can influence patient–provider communication. Filipino patients were described as people who value respect and show respect, especially for older people, and defer to those in authority (e.g., healthcare providers). Filipino patients put a lot of emphasis on communication, interactive engagement, being personable, and establishing a connection, especially with their healthcare providers. The interviewees described that often many family members participate in healthcare decision making, with the oldest family member usually making the final decision. One said, “It’s a family decision, especially medical treatment… It’s usually the oldest that makes that decision” (Rose). Plus, Filipino patients who are not digitally proficient or health literate rely heavily on younger and/or English-speaking family members for help. Interviewees also described that older Filipinos and recent immigrants are usually not familiar with the US healthcare system, may feel uncomfortable seeking care, and need extra support in their care. The interviewees noted that Filipino immigrants may mistrust providers, for example, because of perceived cost. An interviewee noted patients saying things like “‘I don’t want to pay for something I can’t afford’ or ‘I really don’t want to go to hospital. I don’t like doctors.’ There’s always the fear of going to a doctor, going to the hospital. ‘I don’t want to die in the hospital. I don’t! I’d rather die at home’” (Ginger).

#### 3.2.2. Barriers to Effective Communication: Patient, Healthcare Provider, and PMI Levels

LEP Filipino patients were noted to experience multiple barriers to effective communication and interpretation. All interviewees perceived that feelings of shame and pride were Filipino patients’ barriers to acceptance of PMI services. The interviewees described a sense of embarrassment, “linguistic shame” (Basil), and a fear of looking incompetent. “What I found out is that a lot of it is pride. A lot of it is shame. It’s losing face by having somebody to interpret for you, speak for you” (Rose) and “Filipinos have a high pride” (Jasmine). The interviewees described Filipinos as feeling proud of learning English; in addition, English is required for social mobility. Thus, it may be difficult for some patients to admit to not understanding English well. The feeling of embarrassment coupled with the feeling of pride could be the reason some patients overestimate their English skills. One interviewee said, “They think that they [LEP Filipino patients] understood everything, but they did not think about the medical terminology” (Lilly). Additionally, Filipino patients may find it difficult to ask for help and speak up when they need help. The interviewees noted that Filipino patients did not want to appear needful. One said, “They don’t want to be seen as needy or poor, not to have money, and [that they are] not capable” (Rose). The interviewees also noted that some patients may believe that they have to pay for PMI services out of pocket. “Sometimes they’re afraid to ask for interpreters because they [LEP Filipino patients] are worried about the bills” (Basil). Others noted that some patients may refuse PMI services because they trust their family more to interpret, especially if the family member works in the medical field, or if they believe it is more convenient or more common to have a family member interpret for them. However, an issue noted with family interpretation included incomplete or inaccurate interpretation by family member(s). “A lot of times they [LEP Filipino patients] go in [to medical appointments] with their relatives. So, the daughter understood, the son understood, the cousin understood, the sister understood, but they did not try to relay it to the patient” (Fiona). Also, per interviewees, some patients may worry about confidentiality and the disclosure of personal or sensitive information. There is general confusion about the process of PMI services and their benefits. For example, “Because it [PMI service] is not presented to them [LEP Filipino patients] when they check in [for their medical appointment], I think they don’t know that it is even available to them” (Lilly).

Per interviewees, healthcare providers’ barriers to effective communication were limited time and a perceived or actual rushed pace, including fast speech, which Filipino patients could view as a lack of compassion or attention. The interviewees described providers’ fast speech to be a challenge because of the complex and difficult terminology used in the medical field. Additionally, the interviewees had experience with providers who could not accurately assess the need for PMI services, discouraged PMI use, or asked a staff member or a family member to interpret instead of a PMI. One interviewee said, “If they [LEP Filipino patients] can answer ‘yes’ or ‘no’, they [providers] think they speak [English] well. So, they would say, ‘You don’t need an interpreter.’ I encountered two doctors saying that” (Iris). The interviewees also described their concerns related to communication when untrained medical staff act as interpreters, specifically when they have limited proficiency in Filipino languages, a lack of professional interpreting training, and possible feelings of resentment being pushed outside of the official role (job description).

At the PMI level, several personal barriers to medical interpreter employment were noted. The interviewees described barriers such as variability of their income and needing to balance several income sources by choosing between interpretation and other jobs. One noted, “Few [people] want to interpret. There are no incentives. There’s no pension. There’s no 401-K. There’s no health insurance. So why would they [PMIs] [want to] interpret?” (Jasmine). It should be noted that most PMIs in the USA are independent contractors responsible for their own finances, including retirement savings, health insurance, etc. Lastly, the interviewees described medical interpreting as a high-intensity job requiring focused concentration and mental prowess, which can induce stress and fatigue, limiting the number of continuous hours a PMI can work.

#### 3.2.3. Facilitators of Effective Communication: Patient, Healthcare Provider, and PMI Levels

At the patient level, facilitators to effective communication and interpretation included an overall willingness to accept medical care, a previous positive experience with a PMI, and continuity of care with the same PMI. The interviewees noted that LEP Filipino patients tend to understand the value of PMI services after having experienced communication with their provider with the help of a PMI. One interviewee said, “A positive experience will provide future opportunities for families and providers if it’s done properly. That’s why the [PMI] training is very important. It [willingness to accept PMI] can change because they [LEP patient, family, provider] have had a positive experience” (Ginger). The interviewees stated that having an established trust with a healthcare provider also helps improve communication. Plus, having the same sex PMI and healthcare provider may be important for gender-specific care (e.g., gynecology and urology).

Per interviewees, healthcare provider-level facilitators included the ability to recognize language needs as well as health literacy and literacy barriers among their patients. Also, it is helpful when the provider has knowledge of available resources, plans for language services in advance, and can discern when a PMI is needed, such as for important decision-making conversations or surgical consents. Other communication facilitators were providers drawing or using pictures, speaking slower, and using simple or plain language. One interviewee gave an example, “I’ll ask the doctor, ‘Can you please draw the heart and the different valves? Which part of the valve is being replaced?’” (Fiona). The interviewees also noted that providers’ commitment to the principle of benevolence, treating patients with dignity, and following their professional ethics and standards also facilitated effective patient–provider communication.

At the PMI level, the interviewees acknowledged multiple facilitators, such as the importance of strong motivation, commitment, and joy in providing services for those in need. For example, “It’s a personal commitment to really help my community” (Basil), and “…sometimes they [patients] cry too, many tears of joy, meaning ‘Without you [the interpreter], we cannot understand!’” (Cypress). Being personally requested by a patient or provider to return for a follow-up visit resulted in the continuity of PMI work and was perceived as evidence of having performed their work well (facilitating effective patient–provider communication). The interviewees also described the importance of medical interpreter training and knowledge of medical terminology in multiple languages. One stated, “I do tend to interpret word for word. However, there are some [words] that may not exactly translate because of just the idiosyncrasy of the Ilocano language. There might be some English terms that require more of an explanation rather than one word… because of just the way that the language is” (Rose). The interviewees viewed prior experience in the medical field as a facilitator while training in healthcare ethics and standards, professionalism, confidentiality, and neutrality during interactions were noted to be imperative. Other facilitators described the importance of being a “good listener” (Ginger), being patient and flexible, and preparing for appointments. The interviewees believed that PMIs could make appointments more efficient and save time. One interviewee said, “Bringing an interpreter is the key to making it easier for the patient and for the provider” (Mimosa). The interviewees viewed themselves as a “bridge” (Basil and Iris) between American Filipino cultures and patients–providers. For example, “They [LEP Filipino patients] will ask [questions to the provider] because I’m [interpreter] their comfort blanket that knows how to speak respectfully and not to embarrass them…” (Jade) and “They [LEP Filipino patients] will admit something to the interpreter, but they will not admit it to the doctor, like not being able to read and write” (Fiona). All interviewees were Filipino-born emigrants to the USA and believed in understanding Filipino cultural healing practices and being able to facilitate culturally sensitive communications. As one interviewee said, “You [PMI] speak my language—you will understand my [LEP Filipino patient] experience” (Basil). The interviewees helped with patient–provider communication by encouraging providers to slow down and create space for their patients to ask questions. One said, “They [providers] try to understand more, explain more, and spend more time with the patient… when there is an interpreter. They [LEP Filipino patients] talk more or they ask more questions’’ (Iris). Lastly, the interviewees discussed how their role allows the family to be there for support rather than to interpret. “Their [family] role is to be a moral support, to be with their loved one… not to interpret” (Rose) and “I’m here as your [LEP Filipino patient’s] mouthpiece, but also to empower you, to encourage you, and just give you strength to go through whatever it is that you’re going through” (Fiona).

#### 3.2.4. COVID-19 and Remote Interpreting Barriers and Facilitators

Prior to the COVID-19 pandemic, LSH did not offer video remote interpreting and had few interpreting appointments by phone. The interviewees reported that the pandemic reduced their job security because many in-person (face-to-face) appointments were canceled. However, the pandemic also enabled greater availability of remote interpretation through the adoption of new technologies for remote care and health communication. The interviewees had to quickly adapt to video remote interpreting in 2020, and by 2023, video and phone appointments comprised about 20–25% of all interpreting jobs at LSH.

Barriers to remote interpreting included technical difficulties, such as not being able to hear, poor connection, echoes, and the critical need to have stable phone or internet connectivity. Several interviewees said, “Technology is great [only] when it works” (Mimosa and Ginger). It is imperative for all parties to have access to necessary devices (e.g., computer, tablet, and mobile phone) and to know how to use them. Per the interviewees’ experience, this was a serious challenge because some Filipinos did not have access to these technologies or were not proficient at using them. Furthermore, when interpreting over the phone or video, there can be pronunciation issues, making it extra difficult to communicate with patients and medical staff who have heavy accents. The interviewees did not recommend remote communication for older adults and patients with hearing, sight, speech, or other sensory impairments. Overall, interviewees perceived remote types of communication (via phone or video) as more difficult for patients to understand information, less personable, and a barrier for patients to speak directly with their providers. One interviewee said, “In general, I know a lot of patients don’t like the computer. Like I said earlier, I think because it’s not very personable. They [LEP Filipino patients] like to see somebody there [face-to-face]. Some say it is harder to understand [via video]” (Fiona). Plus, remote appointments can add logistical difficulties, such as connecting on time and locating the right department, phone number, and provider. Lastly, remote appointments tend to be canceled more often than in-person.

Facilitators of effective communication and interpretation via remote options included convenience, specifically, not needing to drive and the ability to save time and money on gas. One said, “You don’t have to drive anywhere. If it’s on Zoom, you don’t have to get ready [physically], but I always get ready [preparation for the appointment] no matter what” (Iris). Other benefits included ease for the interpreter to access dictionaries as needed and a greater sense of privacy/anonymity for the patient.

#### 3.2.5. Strengths and Weaknesses of In-Person and Stand-By Interpreting Appointments

Besides remote interpreting, interviewees described other types of appointments to include in-person and stand-by or “shadow” interpreting. Stand-by can be performed either in-person or through video; it is when an interpreter helps only when the patient or provider needs help rather than doing word-for-word interpretation. Eight interviewees estimated that, in their experience, out of 10 stand-by appointments, at least half needed PMI assistance. The interviewees also thought that the stand-by option helped patients realize that they actually needed language assistance, become more accepting of help, and see the value of a PMI for better communication with their providers. The stand-by option was found to be a respectful way of allowing LEP Filipino patients to speak English at their level but ensuring that support was available for them and their healthcare providers on an as-needed basis. Despite that, many providers were not aware of the stand-by interpreting option for a situation when a patient or family member refused an interpreter.

A few weaknesses of the “classic” in-person appointment included transportation challenges and the need to allocate more time and resources for driving, traffic, and gas. The interviewees perceived in-person appointments to strengthen communication because in-person, it is usually easier for all parties (patient, family, healthcare provider, and PMI) to have clear and accurate communication, it is usually easier for PMIs to explain their role, and communication is more personable with greater ability to build rapport. Most interviewees described in-person appointments to be favored over remote by most patients and providers.

#### 3.2.6. Recommendations: System and Provider Levels

At the system level, most interviewees agreed there was a need to better comply with existing Language Access law. From their perspectives, this could be achieved by continuously training more Filipino language speakers to become PMIs through educational and certificate programs. One said, “There should be enough resources to train interpreters, or to make materials out of these [Filipino] languages to make sure that eventually they become available for interpreters” (Basil). The need for additional resources included changes at the state level. “I think we should invest more funding in terms of providing language access for Filipino communities in Hawaiʻi because we are one of the major Asian groups here in the state of Hawaiʻi, and yet, our languages are not that supported” (Basil) and “The whole system of care, it is basically an unfunded law where organizations have to find ways to put it in their budget somehow” (Rose). One interviewee said, “You gotta make an incentive for providers. You gotta make it attractive to them to be able to provide it [PMI services]” (Rose). A recommendation for hospitals was to consider having more in-house interpreters, specifically for Filipino languages. A recommendation for language services providers (e.g., LSH) was to assign the same interpreter to a particular patient, if possible, for continuity and rapport. The interviewees stressed the importance of a culturally trained healthcare workforce. They recommended healthcare systems provide CLAS training for providers, administrators, receptionists, and all staff who engage with patients. In addition, hospitals should have an effective system to better identify patients in need of language services, including educating patients about the availability of and their right to ask for free interpreting services. The last recommendation was to increase public awareness of this right. The interviewees suggested encouraging local Filipino community leaders to aid in raising awareness utilizing the Filipino Community Center (“FilCom”), social media, Filipino radio and TV stations, and churches.

For providers, the main recommendation was to support PMIs and to avoid using ad hoc interpreters (family members or untrained staff) as they might not have the skills necessary for interactive, critical, or complex medical conversations. The interviewees agreed that ad hoc interpreters should be used only in emergencies or for basic, comforting conversations (e.g., helping a patient to the restroom). Additionally, providers should be mindful of the health literacy and literacy needs of their LEP Filipino patients and explain information in ways that can be understood, for example, with pictures or using simpler terms. The interviewees noted that providers should slow down when speaking, listen, and give patients time to speak. Lastly, the interviewees suggested that healthcare providers should be offered or required to complete continued education about CLAS, language access resources, the role and importance of PMIs, communication techniques, and the potential bias that may occur when family members are used as interpreters.

## 4. Discussion

The purpose of this study was to explore perceived barriers and facilitators to providing CLAS for Hawaii’s Filipino LEP patients from the perspective of PMIs. Interviewees described context-specific factors that influence patient–provider communication and barriers and facilitators to CLAS provision. They also provided recommendations about ways to improve CLAS provision.

This study found that LEP Filipino patients in Hawaiʻi have been underserved. Based on the quantitative survey, the majority of PMIs for Filipino languages in the state are older and do not work full-time. A total of 9 out of 10 interviewees agreed that Hawaiʻi needs more interpreters for Filipino languages, and 8 out of 10 agreed somewhat or agreed completely that there were health appointments that occurred without a PMI present while a PMI was needed (this perception was based primarily on what PMIs heard from their clients). The LEP Filipino community in Hawaiʻi could benefit from learning more about their language access rights.

To our knowledge, no prior studies have examined the perspective of PMIs for Filipino languages, but the findings of this study are supported by other research on the topic. In general, patients with LEP are at risk of being under informed and overwhelmed by the large volume of medical information given over a short time [44]. This study highlighted the role of PMIs as change agents for more accurate and effective patient–provider communication, especially among recent immigrants and older people. Other research supports our findings that poor health literacy and difficulties communicating health needs are common among immigrants or refugees [50]. We found that for LEP Filipino patients, PMIs act as cultural bridges/liaisons and health literacy/literacy guardians that help establish accurate and effective communication, trust, and rapport with their providers. PMIs tailor interpreting in a culturally sensitive manner, encourage providers to slow down and use simpler language, and empower patients to ask questions and be active participants in their medical care. This is in agreement with previous research reporting that in communication with LEP patients, it is important to build trust and to have time for patient education and support [47,48,50,51]. LEP patients benefit from interpreter services because PMIs can function as cultural brokers and literacy guardians, which is especially important in complex medical care and for medical decision making [49,51]. PMIs also support communication through cultural sensitivity, clarification of information, and asking providers to adjust to patients’ health literacy levels [26,47]. Providers and staff could improve communication with LEP patients by communicating with cultural competence, checking a patient’s language (dialect), checking for patient understanding, and using simpler language and shorter sentences [26,47]. Similar to other research, our study found that PMIs uphold medical interpreting standards and strive to convey information accurately, confidentially, and impartially [26,34]. Other studies also found potential barriers to effective patient–provider communication with PMI’s assistance to include perceived sensitive information sharing if a PMI knew a patient in the community, the lack of continuity of interpretation with the same PMI, and sometimes family involvement [26,51]. Although both trained bilingual health staff and PMIs can be interpreters, their roles are different, and it is important to recognize the appropriate use of each [46,47,57]. Our study found that PMIs, rather than family members, should be interpreting and that providers should collaborate with and support the use of PMIs. This is supported by previous research that reported better service for LEP patients when PMIs and not family members are utilized, which can be achieved through improved access to PMIs and system-level changes [46,47]. We found that LEP Filipino patients prefer more in-person and person-to-person connections. Other research supports that, compared to remote interpretation, in-person or face-to-face communication was more effective for more nuanced and interactive conversations and for facilitating cultural understanding [47,59]. Lastly, our study fundings were consistent with other studies about the impact of the COVID-19 pandemic on PMI services. Overall, the pandemic led to a decrease in PMI utilization [53]. However, while in-person interpreting appointments decreased, PMIs noticed more remote interpreting appointments and the need to adopt remote communication techniques [54,55,56].

PMIs are necessary for equitable healthcare because LEP patients should be able to understand communicated medical information the first time they encounter it. Healthcare organizations have a legal responsibility to provide information in a manner appropriate for all their audiences [30]. This can be achieved by following the Joint Commission requirements for advancing effective communication, cultural competence, and patient- and family-centered care (the National CLAS standards) [60]. Hospitals should be offering language assistance and ensuring the competence of individuals (bilingual health staff) providing language assistance [13]. PMIs are trained to provide such language services. In interactive medical conversations, a communication triad should include the LEP patient, clinician/provider, and PMI.

### Limitations and Future Research

While this study had many strengths, certain limitations and biases should be acknowledged. For instance, results may have been impacted by self-selection bias since involvement was voluntary. In addition, the use of interviews may have led to participants answering questions in ways that presented them in a more socially acceptable perspective. Moreover, all participants were recruited from a single community partner and worked for the same language services provider. This may have limited the generalizability of our findings, and since the study was focused on LEP Filipinos in Hawaiʻi, there could potentially be less generalizability to Filipinos living in other US states. The study is also limited by its specific focus on PMIs and their perspectives on the facilitators and barriers to effective communication. Although all interviews were extensive in length, with expert informants recruited via purposeful sampling, our sample size was limited. However, this can also be viewed as a strength of the study because of the focus on the perspectives of understudied PMIs’ Filipino languages. Plus, all interviewees were immigrants to the USA from the Philippines; thus, they had personal expertise and cultural competence. Research shows that as few as four expert informants can be sufficiently informative to develop a theory [61], and data saturation can be reached after six interviews in nonprobabilistic, purposeful sampling [62].

Future investigations should strive to create a more comprehensive understanding of the challenges to the provision of CLAS by garnering perspectives from patients, family members, healthcare providers, stakeholders, and leaders. Future intervention work may involve Filipino community leaders and organizations who strive to help educate the populations they serve about language access rights in healthcare settings. Other intervention work should include strategies to improve patient–provider communication with LEP patients. Furthermore, future research should also address other popular languages in Hawaiʻi, including Korean, Mandarin, and Chuukese (Micronesian language), among others.

## 5. Conclusions

Language assistance is a patient’s right and not an option. The use of PMIs meets the legal requirements of Title IV of the Civil Rights Act of 1964 and should be offered to LEP Filipino patients. This study highlights the fact that there is an unmet need for language assistance for LEP Filipinos in Hawaiʻi. There appears to be a need for more resources and funding to adequately fulfill the Language Access law in the state of Hawaiʻi.

To advance health equity, it is important to ensure LEP Filipinos are aware that language assistance via PMI is their right and is provided to them at no cost. At the system level, there should be more CLAS training for the healthcare workforce, and at the practice level, there should be more support for the consistent use of PMIs and not family or untrained individuals. PMIs should be included in all interactive medical conversations with LEP Filipinos. When LEP patients or their family members refuse PMI support, a stand-by option should be provided.

## Figures and Tables

**Table 1:** Examples of interview questions by domain of influence.

**Interpreter level**
- Tell me about your typical day working as a medical interpreter.
- Describe the training you completed to become a medical interpreter.
- What does effective patient-provider communication mean from your point of view?
- What do you do to help patient-provider communication?
- Who are the most typical patients you interpret for (e.g., elderly, new immigrants)?
**Patient and family level**
- Do you think Filipino patients speak up if or when they need an interpreter?
- What do you think helps Filipino patients with LEP to express themselves better?
- Do patients ever request a language other than their mother tongue (main dialect/language)?
- What would make a Filipino patient with LEP not want an interpreter?
- What role does Filipino culture play in medical settings and decision making?
- Describe the role of the family in medical interpretation for Filipino patients.
- How might family facilitate or be a barrier to effective communication?
**Provider level**
- What do you think helps providers to communicate better with Filipino patients?
- What do you think a provider should know about Filipino LEP patients?
- Have you seen medical staff act as an interpreter? Are there situations when it is ok?
- Do you think providers may assume an LEP Filipino patient does not need an interpreter when the patient needs one? If yes, why?
- Have you noticed a preference among providers for in-person or remote interpreting?
**System and practice level**
- What do you think in the medical setting could be done differently to make medical interpretation more accessible and responsive to patient needs?
- Describe how the infrastructure (internet connectivity, layout, unit setting) of different medical facilities influences accessibility and quality of medical interpretation.
**COVID-19 pandemic**
- How do you think the COVID-19 pandemic impacted the use of medical interpreters for patients/providers/organizations (e.g., hospitals)?
- How might Filipino culture or background influence the adoption or use of technology (e.g., telehealth) for communication with health providers?

**Table 2:** Survey results *.

Characteristics	Mean ± SD (Range)	*n* (Percent)
Age	55.1 ± 11 (32–67)	
Number of years lived in the USA	36.2 ± 15 (10–53)	
Number of years lived in Hawaiʻi	31.1 ± 16.2 (10–53)	
Years of experience as PMI	8.1 (5 months–24 years)	
Average number of PMI work hours per week	10.3 (2–30 h)	
**Sex**		
Female		8 (80%)
Male		2 (20%)
**Race/ethnicity**		
Filipino		10 (100%)
**Country of birth**		
Philippines		10 (100%)
**Education**		
Associates		4 (40%)
Bachelors		4 (40%)
Doctorate		2 (20%)
**Country(ies) education received**		
Philippines		1 (10%)
USA		4 (40%)
Philippines & USA		4 (40%)
Philippines & USA & UK		1 (10%)
**Medical interpreter training**		
One-on-one training by experienced interpreter		1 (10%)
Interpreting classes		2 (20%)
Interpreting classes & Interpreting workshops		1 (10%)
Medical classes & Interpreting classes & Interpreting workshops		1 (10%)
Medical classes & Interpreting classes & One-on-one…		1 (10%)
Medical classes & Interpreting workshops & One-on-one…		1 (10%)
Medical classes & Interpreting classes & Interpreting workshops & One-on-one…		3 (30%)
**Interpreting languages**		
Ilocano		1 (10%)
Ilocano & Tagalog		4 (40%)
Ilocano & Tagalog & Cebuano		1 (10%)
Ilocano & Tagalog & Pangasinan		1 (10%)
Tagalog & Cebuano		1 (10%)
Tagalog		2 (20%)
**Appointment types experienced in**		
Phone		1 (10%)
Phone & Video		1 (10%)
In-person & Phone & Stand-by		2 (20%)
In-person & Video & Phone & Stand-by		6 (60%)
**In your opinion, we need more trained Filipino interpreters in Hawaiʻi…**		
Agree completely		9 (90%)
Disagree somewhat		1 (10%)
**In your opinion, there are many healthcare appointments when an interpreter was needed, but none was provided…**		
Agree completely		5 (50%)
Agree somewhat		3 (30%)
I do not know/no opinion/no experience		2 (20%)

* Sample size = 10 (right column). Survey results include a listing of participants’ demographic characteristics, work experience, medical interpreter training, Filipino languages of interpreting, experience with different appointment types, and responses to Likert scale questions.

## Data Availability

The data presented in this study are available on request from the corresponding author. The data are not publicly available due to privacy concerns of interview participants.

## Appendix A

**Table A1:** Additional illustrative quotes for the six themes.

**1. Cultural and Social Factors that Can Influence Patient–Provider Communication**
“It may take time for the new arrivals, the new arrived families to assimilate better…To me, it’s more like because they’re [elderly Filipino LEP patients] embarrassed and shy and they can’t really assimilate with the accent and the English and they’re hard of hearing.” (Jade) “The family is expected to be part of the treatment. If there’s treatment involved, the family is expected to help to make sure that the treatment is done or supported.” (Rose) “Paternal, the oldest, whoever’s the father. Whoever’s the matriarch if there’s no patriarch, the maternal matriarch. If they’re not available, it’s whoever is the oldest person. It just depends. There’s a lot of politics involved with the family.” (Ginger) “It’s rude for the doctor, a lot of times, they’ll just say their [older Filipino patients] first name… But they’re younger than the patient, and that’s rude in Filipino culture.” (Fiona)
**2. Barriers to Effective Communication: Patient, Healthcare Provider, and PMI Levels**
Patient Factors
Shame, Embarrassment, and Pride “The thing is the embarrassment of not being more prepared communication wise.” (Jade) “Shame. In the Philippines, asking for help is a sign of weakness. Asking for help, asking for somebody to explain, to speak on their behalf, is a sign of weakness… in the Philippines, as long as you’re capable, you’re able to talk and walk…So, when asking for help here, especially free services or government services, they have a lot of reservations in asking or willing to accept help.” (Rose) “When you ask something to a Filipino, they will say, ‘Yeah, yeah.’ But actually, they did not understand 100% totally. Because within themselves, when you think of them that their second language is English, they want you to know that they understand more. But, actually, it’s not. Because within themselves they have that pride. It’s a custom.” (Jasmine) Cost “I think part of that also is because patients think that they have to pay for it [PMI services].” (Fiona) “If they [LEP Filipinos] were aware that there is that [PMI] service, they would definitely perhaps say, ‘Yes, I need an interpreter.‘ But, many Filipinos, especially the immigrant Filipinos, are not aware that you [patient] can even hire an interpreter and there is also this factor of the fear to pay.” (Basil) English Proficiency and Literacy “[There is] a handful [of patients] who cannot read and write. I’m helping somebody right now who cannot read and write. And that is hard for them to admit.” (Fiona) “I mean, they [Filipino patients] do not go to their appointments because of the fear of not understanding the provider. So, a lot of Filipinos won’t go to [medical] appointments because of the language barrier.” (Lilly) “Because a lot of the patients I’m helping conversation wise, they think they speak it [English] well, the language, but then when it comes to medical terms or when it comes to having an appointment, they cannot express themselves.” (Iris) Family “And it gets complicated, and it becomes political within the family. You need a third-party interpreter who is certified to make sure the complete translation [interpretation] goes between the provider and the patient, and there’s no miscommunication.” (Ginger) “And so that was very difficult because she [mother] needed me as her daughter, and she also needed an interpreter, and I was serving as both. And then towards the end, after so many sessions, she said, ‘I need my daughter. I don’t want a nurse. I want my daughter now.’” (Rose) “We [Filipinos] are a patriarchal society. Especially Filipino men, they’re not used to seeking help.” (Basil)
Provider Factors
“There are very few doctors that will say they [patients] do not need one [interpreter]. ‘Why do you have an interpreter? You speak English fine.’ And that is irritating, and that is annoying. You [provider] should be also supportive of us [PMIs] because I clearly know that she [patient] is not understanding everything you are saying. I know that for a fact because she [patient] is not answering you properly.” (Fiona) “Sometimes the doctors or the nurse would just talk to me rather than talking to the patient, when in fact, they’re the ones who are supposed to be talking, and I’m just supposed to be bridging the two of them. But by doing that, the doctor is *invisibilizing* the patient.” (Basil) “Their language [Filipino nurses] is conversational…some of the nurses that I talked to, they’re like ‘I do not know what body part that is.’ I mean, they didn’t know how to say it in Ilocano or Tagalog.” (Fiona)
PMI Factors
“If you had to support a family, you can’t do this job. First of all, I do not think that it would be feasible because you would be driving all over the place to really make eight hours. And that’s every day and driving through traffic. It would have to be back, to back, to back, and that is stressful.” (Fiona) “Yeah, I don’t know how the scheduling would work because then that would interfere, unless you just get called in on your days off. The ones that I’ve talked to about [interpreting], they’ve mentioned wanting to become an interpreter but after retiring.” (Fiona) “You could be making more working at Costco or Safeway but with benefits.” (Rose)
**3. Facilitators of Effective Communication: Patient, Healthcare Provider, and PMI Levels**
Patient Factors
“If you are doing a great job for the patient, the provider will recommend you every time or ask you what your availability is to go back there.” (Cypress) “If they [family members] sense that you’re friendly and they sense that you’re helpful to their family members, the patient, then they request you again. They see our worth as an interpreter, that we are helpful and friendly, that their folks or their parents are comfortable with an interpreter.” (Lilly) “But if they feel it is very personal, females will request female interpreters and probably a female provider, as well as males. Prostate cancer [patients] prefer a male interpreter and a male provider.” (Ginger)
Provider Factors
“I like the physicians where they know how interpreting works: ‘I [physician] say something. Interpreter says something.’ And some are even really strict: ‘You can only speak in Ilocano and Tagalog. Otherwise, what’s the point of having an interpreter if you’re going to try to speak in English?’ Some doctors are really good at just using the interpreter. That helps a lot.” (Fiona) “The doctor can show the drawing for the parts of the body, like colon, and then they [patients] can understand.” (Mimosa) “I like to ask them [health care providers] to point at pictures. I will actually ask the doctor if they have a chart if it’s like a biopsy of something, and they have charts or models of intestines and stuff. ‘Doc, can you just show which part it is exactly?’ So, they’ll gladly draw and then that simplifies it for the patient.” (Fiona)
PMI Factors
“And you have to have the heart to do it. You can’t just interpret because you speak Ilocano or Tagalog. Or maybe you have a medical background, and you speak the language that you can interpret. No, you have to have the heart and the passion to actually help people with their mother tongue.” (Lilly) “I always think of the context, the culture. It’s like always hovering around me when I’m doing interpretation because when I’m sensing that this person is not getting it, then I will have to find a way to approach it from a more cultural approach to make sure that this person gets it. And then when he articulates a response or reply, I also have to find a way to make sure to articulate that to the doctor…” (Basil) “I think maybe without an interpreter, the doctor could just easily spew out all kinds of medical terms, all kinds of different words and big words, and the patient would be okay with that. So having an interpreter there, he [doctor] has to be more conscious of making it in layman’s terms, simpler.” (Fiona) Effective communication is when… “They [patients] can make the right choice medically.” (Jade) “The patient will not be nervous. Patient can tell everything.” (Cypress) “When the patient asks the right questions, or the patient is responding relevantly.” (Lilly)
**4. COVID-19 and Remote Interpreting Barriers and Facilitators**
Barriers “We have so many dialects or languages in the Philippines that weren’t there [video interpreting platforms]. I ended up having to interpret the majority and not Martti. We just turned off Martti because it wasn’t working.” (Rose) “Sometimes it [remote] doesn’t work on their phone because they don’t have a smartphone and that patient probably doesn’t own an iPad or a computer or a laptop.” (Lilly) “Sometimes the videos, what I hear from the patients, it’s harder for them to explain and it’s harder for them to ask the person on the video questions. Especially for the older ones that have hearing impaired. I mean they could barely hear you in-person already.” (Fiona) Facilitators “I guess people were scared to have an appointment, to go and see a doctor. That’s why good thing they came up with a Zoom meeting because we didn’t really do much on Zoom before.” (Iris) “They [patients] don’t have to jump in the car or take the bus. And for some, they feel safer than in terms of not being exposed to potentially Covid. So, there’s some benefits to using technology or telehealth.” (Rose) “I think [access to PMIs] improved because of technology. Now, we have learned how to use the technology, how to make it work for us.” (Rose)
**5. Strengths and Weaknesses of In-Person and Stand-By Interpreting Appointments**
In-person
“When you [PMI] are there in-person, you get to feel everybody in the room. So, you know when to answer or interpret, and it’s different. It’s a different feeling when you’re in-person. The patient feels more comfortable, so they open up more… A lot of patients prefer in-person over the phone.” (Lilly) “Yes, I think providers like more in-person, the quality of conversation, so that it will deliver directly to the patient what’s going on or what the doctor is doing or why the patient is in their office.” (Mimosa) “I prefer in-person to be honest with you… You cannot see their [patients and providers] faces. A lot of times they [patients] may not be able to find the words but their face tells it all… You can’t really have those same elements in a video.” (Rose)
Stand-by
“So, when that happens [patient refuses PMI], I’ll just say, ‘Okay, I’m already here; I’ll just go in with you and if you need help, I’m here. If you don’t need help, then that’s fine too. But I’ll just sit right here’… So, I’ll let her [patient] answer as much as she can. And if the doctor looks at me, then I’ll explain what the doctor said, or maybe repeat what she said.” (Fiona) “So, I would say, ‘I’ll be outside’ or ‘I’m here, if you need me.’ And then later on, the nurse would come and get me if the patient needs help.” (Iris) “When I’m there for the appointment, and then she [patient] said she was okay, yet in the middle of the appointment, there were words that she didn’t understand. So, the stand-by becomes an interpreter full time.” (Jasmine)
**6. Recommendations: System and Provider Levels**
System level: Policies and Procedures
“We [Filipinos] are the second or third largest population in Hawaiʻi. You’re going to need them [PMIs]. And usually, demand is when there’s a need… because there is a large population that comes from the Philippines all the time, they will have the need.” (Ginger) “If they knew that it [PMI language access] is a service and it’s for free, I don’t think they [patients with LEP] would resist… Explain that they don’t have to pay for it. It’s not their insurance. It’s not included in their co-pay. It’s a service that the hospital is providing for you [patient] at no cost.” (Fiona) “Department of Health uses Language Services Hawaiʻi because it’s federally funded, like the WIC program is a federally funded program. So, they are required to provide language access to their clients. So that’s budgeted. That seems to work.” (Rose)
Provider and Practice Level
“I’ve seen it when nurses interpret and they’re not really doing interpretation. They’re basically telling the patient as far as what’s happening. They’re not interpreting. They’re crossing the line basically… inserting their own opinion or analyzing and evaluating and all of that stuff that they do as nurses, and they are not interpreting word for word.” (Rose) “We [providers and interpreters] have to speak in simple terms, even simpler than third grade. I know, that’s the standard, but I think simpler than that. And if we could use more visuals, that’s also very helpful… When they [providers] are explaining something, just get the pencil and pads and start drawing stuff rather than just speaking… We need to make sure that we use visual tools more, especially with limited English proficiency.” (Rose) “But when it comes to life and death decisions, it becomes a little bit more emotional. You really need an interpreter. You need someone who is not related, a third party to interpret, because your emotions get in the way and the interpretation gets miscommunicated.” (Ginger)

## Appendix B

Additional illustrative stories and examples from PMIs.

- “In the Filipino culture, we hold our hand and put it on our forehead. And they [older Filipino patients] are more comfortable [when] doing that. And then they say, ‘I have a son.’ They [older Filipino patients] called me [PMI] son because of the respect that I showed to them. That is the culture of Filipino people. Then they are more comfortable.” (Cypress)
- “He [PMI’s Filipino family member] looks forward to seeing him [doctor] because he [doctor] shakes his hands, and he calls him Mr. [last name]. ‘How are you, my friend? What’s going on? What are you doing?’ Those kinds of personal conversations. And then he [doctor] goes into, ‘This is your blood pressure.’ And it doesn’t take that much. It only takes a few seconds to say, ‘How are you? What did you eat today? How was yesterday?’ Have those personal conversations initially, and then at the end of the medical appointment, say, ‘Thank you for being here. We’ll see you in six months. You’re doing well, we’ll see you in six months, keep it up.’ Those kinds of encouragements.” (Rose)
- “Especially the kidney transplant—the provider discovered there was abuse because there was no family. They will always ask, ‘Who is going to help you to care for yourself? Is there somebody? We need to know that family, they need to come, and if there’s none, how can we help?’ So, that’s very important because they do not want the patient to have the surgery, and all of a sudden, it failed because there was no one taking care of them physically—not so much as moral, but also physical because after surgery on diabetes or kidney transplant, it is very critical.” (Jade)
- “I think it’s best to just have the patient, the interpreter, and the doctor [and no family] so they are able to make decisions for themselves… I’ve seen situations where the patient really wants to, but then the daughter is insisting, ‘I think you should wait.’ But I know for a fact that the mom wants to have the procedure done because she is not comfortable, it [disease] is giving her a hard time. She really wants to do it [surgery]. And so, she postponed it several times because the daughter is like, ‘No, we are gonna just postpone it again, and see how you feel the next time.’ They [providers] have postponed it several times. But I bet you if it was just the patient, me, and a doctor, she [patient] would have convinced the doctor to do it, and the doctor would have already done that procedure.” (Fiona)
- “Religion is also a big factor in Filipino families. For instance, there are certain cases when somebody needs medical help or is sick, but then your parents would say, ‘Just pray. You will feel better.’ And it has happened so many times, and I have seen this so many times, that the parents, or the elders, or the auntie would say, ‘You just need to pray to be better or to actually be cured.’ But then we know that prayers are actually not enough if you need medical attention.” (Basil)
- “For me, there’s just pure genuine joy that you [PMI] are able to speak in your language and help them [patients] articulate those [medical] things so that they feel safe. At the same time, they feel that they are provided with the right services that they need urgently or at the moment. And sometimes even they [patients] feel that a family is there with them. One time I was doing a medical interpretation for this old man, and he had a heart [condition or] something. He was alone. So, his child was not there, and his child grew up here, so the child speaks to him in English. He [patient] can understand but he said that it is completely different when somebody was there for him speaking in Cebuano and then interpreting for him in English because he said as if somebody was present like a family.” (Basil)
- “He [LEP patient] speaks Ilocano and the interpreter on iPad was Tagalog. And that person [patient] did not say anything. Again, Filipinos do not speak up. He [patient] did not say anything. And then later on, he told me at the next appointment, he [patient] did not understand anything because he spoke Ilocano and he was given Tagalog [interpreter] on the iPad.” (Iris)
- “For example, a patient that I helped who was pregnant. And then while pregnant, her husband had an affair. So, then they asked questions like, ‘Are you safe in your relationship? Are you sad? Are you depressed? Anxiety?’ and so on. Then she said, ‘I’ve been just kind of depressed.’ So, I asked, ‘How come?’ She said, ‘My husband had an affair. It’s been so hard.’ And she just had a newborn baby. And so, I asked her, ‘Is it okay for me to share with the nurse and the doctor so that they know how to care for you?’ And she said, ‘Yeah, that’s fine.’ Now the doctor said, ‘We need to get you tested for STD. We need to get you a pregnancy test.’ So now they can take care of her. If she had not shared that, because I didn’t have the connection with her, I would not have known any of that.” (Fiona)
- “Doctors like assuming everybody knows what depression with hallucination is without explaining. I [interpreter] can’t explain that if they are [doctors] not explaining it because I’m to interpret only what comes out of the doctor’s mouth. The doctor has been thinking the patient is experiencing hallucinations. So further probing I asked [the patient], ‘Do you know what hallucinations mean?’ Of course, I asked the doctor’s permission, ‘Can I ask her if she knows what hallucination means?’ And so, the doctor said, ‘Sure, please do.’ And so, she said, ‘I hear voices,’ but it is mostly thoughts that she was thinking. She was not hearing voices! So, they’ve been giving her hallucination drugs, medicines. So, the doctor was like, ‘Oh no. I was told that she has hallucinations.’” (Fiona)
- “I don’t think that they [patients] understand fully. Like this one patient that I was helping, the cancer came back and then he was offered chemotherapy after the surgery. And he declined it. So now, cancer came back, and doctors told him because you did not [do chemotherapy], this was the chance you took. You declined chemo at the time and so cancer came back. I don’t think it was explained properly because he said, “It was suggested, but it was never explained. Nobody ever called me back and so I did not know what it was all about!” He did not know what chemotherapy was. He did not know what the purpose in all of that was.” (Fiona)
- “There’s one doctor that I really like working with because she’ll ask and request an interpreter, regardless of if you bring a relative. Because she’s had an experience. One time she said she had to bring back the patient again, because the first time around, she brought her son. And so, the doctor asked the son, ‘You’re here to help your mom so can you interpret for your mom? Repeat everything I just said in Ilocano to your mom.’ And so, the son couldn’t repeat it, couldn’t explain it to the mom. I think that was an eye opening for the physician. She will have an interpreter every single time if they speak Filipino, Ilocano.” (Fiona)
